# In vitro selection of *Plasmodium falciparum Pfcrt* and *Pfmdr1* variants by artemisinin

**DOI:** 10.1186/s12936-016-1443-y

**Published:** 2016-07-22

**Authors:** Muturi J. Njokah, Joseph N. Kang’ethe, Johnson Kinyua, Daniel Kariuki, Francis T. Kimani

**Affiliations:** Jomo Kenyatta University of Agriculture and Technology (JKUAT), P.O. Box 62000-00200, Nairobi, Kenya; Kenya Medical Research Institute (KEMRI), Centre for Biotechnology Research and Development (CBRD), P.O. Box 54840-00200, Nairobi, Kenya

**Keywords:** Artemisinin, Drug resistance, Malaria

## Abstract

**Background:**

Anti-malarial drugs are the major focus in the prevention and treatment of malaria. Artemisinin-based combination therapy (ACT) is the WHO recommended first-line treatment for *Plasmodium falciparum* malaria across the endemic world. Also ACT is increasingly relied upon in treating *Plasmodium vivax* malaria where chloroquine is failing. The emergence of artemisinin drug-resistant parasites is a serious threat faced by global malaria control programmes. Therefore, the success of treatment and intervention strategies is highly pegged on understanding the genetic basis of resistance.

**Methods:**

Here, resistance in *P. falciparum* was generated in vitro for artemisinin to produce levels above clinically relevant concentrations in vivo, and the molecular haplotypes investigated. Genomic DNA was extracted using the QIAamp mini DNA kit. DNA sequences of *Pfk13, Pfcrt* and *Pfmdr1* genes were amplified by PCR and the amplicons were successfully sequenced. Single nucleotide polymorphisms were traced by standard bidirectional sequencing and reading the transcripts against wild-type sequences in Codon code Aligner Version 5.1 and NCBI blast.

**Results:**

Exposure of parasite strains D6 and W2 to artemisinin resulted in a decrease in parasite susceptibility to artemisinin (W2 and D6) and lumefantrine (D6 only). The parasites exhibited elevated IC50s to multiple artemisinins, with >twofold resistance to artemisinin; however, the resistance index obtained with standard methods was noticeably less than expected for parasite lines recovered from 50 µg/ml 48 h drug pressure. The change in parasite susceptibility was associated with *Pfmdr*-185K mutation, a mutation never reported before. The *Pfcrt*-CVMNK genotype (*Pfcrt* codons 72–76) was retained and notably, the study did not detect any polymorphisms reported to reduce *P. falciparum* susceptibility in vivo in the coding sequences of the *Pfk13* gene.

**Discussion:**

This data demonstrate that *P. falciparum* has the capacity to develop resistance to artemisinin derivatives in vitro and that this phenotype is achieved by mutations in *Pfmdr1*, the genetic changes that are also underpinning lumefantrine resistance. This finding is of practical importance, because artemisinin drugs in Kenya are used in combination with lumefantrine for the treatment of malaria.

**Conclusion:**

Artemisinin resistance phenotype as has been shown in this work, is a decrease in parasites susceptibility to artemisinin derivatives together with the parasite’s ability to recover from drug-induced dormancy after exposure to drug dosage above the in vivo clinical concentrations. The study surmises that *Pfmdr1* may play a role in the anti-malarial activity of artemisinin.

## Background

Malaria is one of the most serious life-threatening protozoan diseases, classically identified by paroxysm, fever, and flu-like symptoms recurring in 48- or 72-h cycles. The World Health Organization (WHO) recommends artemisinin-based combination therapy (ACT) as the first-line treatment for falciparum malaria in all endemic regions [1]. There is already evidence of reduced susceptibility of *P. falciparum* to artemisinin derivatives in Southeast Asia which is manifested by the delayed parasite clearance times in vivo [2, 3]. WHO has launched an action plan to stop possibly emerging resistance at an early stage so as to protect ACT [4].

The *P. falciparum* multidrug resistance 1 gene (*Pfmdr1*), *P. falciparum* chloroquine resistance transporter gene (*Pfcrt*), and *kelch13* propeller region (*Pfk13*) gene single nucleotide polymorphisms (SNPs) are believed to be markers of resistance to a great number of anti-malarial drugs, including ACT [5, 6].

Increased copy number and sequence variation in *Pfmdr1* at the amino acid positions N86Y, S1034, N1042 and D1246Y have been reported to interfere with the parasite susceptibility to dihydroartemisinin [7], artemisinin [8, 9], mefloquine [10], quinine [8], halofantrine [11], piperaquine [9], lumefantrine [12], amodiaquine [13] and chloroquine [14]. Recent in vitro work have strongly linked *Pfmdr1* to drug transportation [15].

The *Pfcrt* protein has capacity to efflux peptides and glutathione [16], and transport anti-malarials besides chloroquine [17]. Its codon K76T mutation has been linked to the development of chloroquine resistance [18]. *Pfcrt* SNPs have also been found to be associated with the in vitro and/or in vivo parasite susceptibility to artemisinin [19], lumefantrine [20], quinine [21], mefloquine [18] and piperaquine [22].

The *Pfk13* SNPs;C580Y, R539T, I543T, and Y493H were the four main alleles which were observed to be significantly involved in artemisinin resistance out of the 17 non-synonymous SNPs found in Cambodia [5]. Other genes associated with artemisinin resistance include; *Pfcrt* [23]*, P. falciparum* translationally controlled tumor protein (*Pftctp*) gene [24]*, Pfmdr1, P. falciparum* multidrug resistance-associated protein-1 (*Pfmrp1*) [9] and ABC transporters [25]. Also there are target proteins; *P. falciparum* calcium adenosine triphosphatase 6 (*Pfatpase6*) [26] and *P. falciparum* ubiquitin c-terminal hydrolase (*Pfubcth*), the orthologue of *P. chabaudi* ubiquitin protein-1 [27].

The identification of the genetic determinants will be cornerstone for understanding the molecular basis of artemisinin drug resistance and will, therefore, provide basic evidence for molecular surveillance of drug-resistant *P. falciparum* strains for epidemiological surveys. This study reports an assessment of anti-malarial resistance marker polymorphisms in *Pfmdr1*, *Pfcrt*, and *Pfk13* genes in chloroquine resistant W2 and chloroquine sensitive D6 lines cultured in presence of artemisinin.

## Methods

### Study site

The study was carried out at the Centre for Biotechnology Research and Development (CBRD) malaria laboratory, Kenya Medical Research Institute (KEMRI) Headquarters, Nairobi, Kenya.

### Parasite cultures

Two *P. falciparum* laboratory clones, W2 (Indochina) and D6 (Sierra Leone) were cultivated by standard methods, as described by Trager and Jensen [28] with some modifications described by Benoit-Vical et al. [29]. Continuous routine cultures were maintained at 1–5 % parasitaemia in complete medium containing 12.5 % heat inactivated human serum (blood type A, AB and O pooled) and in O^+^ human erythrocytes. Haematocrit was adjusted to 6 % O^+^ type RBCs of the 5 ml total culture volume. The choice of the two parasite lines (D6 and W2) was made in the anticipation that different genetic backgrounds of the parasites may provide a wider span of genetic determinants during the in vitro evolution by artemisinin selection.

### Development of resistant parasite lines

Artemisinin resistance was induced in *P.falciparum* W2 and D6 lines as described by Oduola et al. [30]. *Plasmodium falciparum* W2 and D6 lines were cultured under increasing artemisinin pressure in stepwise increments over 450 days. The starting concentration of artemisinin was 10 µg/ml for the two parasite clones (W2 and D6). Then, increments were in escalations of 10, 20, 30, 40 and 50 µg/ml, with an exposure time of 48 h at each increment.

The drug pressure cycle started with the addition of artemisinin to asynchronous cultures with 4–8 % parasitaemia. The 12.5 % human-serum-supplemented growth medium (RPMI Medium 1640; Gibco^®^), with or without artemisinin, was changed after 24 h. The artemisinin pressure was removed after 48 h, the time at which parasite morphology was degraded and growth was significantly reduced. The medium containing artemisinin was discarded and parasites washed twice with drug free wash media. Then, drug-free culture medium supplemented with 12.5 % human-serum was added to cultures, which were incubated at 37 °C until parasite growth and morphology became normal.

A parasite was considered “tolerant” to a particular artemisinin concentration when the recovery phase occurred within 48 h. The artemisinin exposure time was between 42 and 173 days so as to reduce the recovery time to below 48 h for the W2 and D6 lines at each increment. This was considered as a cycle of artemisinin pressure. At this point, artemisinin pressure was resumed with the consecutive higher artemisinin concentration in the increment to culture which has attained ≥5 % parasitaemia. During adaptation, parasite was frozen and the procedure was resumed from the cryopreserved stocks to maintain this phenotype.

Bulk culture of W2 cycle 5 culture A at 50 µg/ml artemisinin (W2 c5A), D6 cycle 3 culture A at 30 µg/ml artemisinin (D6 c3A) and D6 cycle 5 culture A at 50 µg/ml artemisinin (D6 c5A) parasites was cloned by limiting dilution for 35 days to give W2 cycle 5 sub-culture B (W2 c5B), D6 cycle 3 sub-culture B (D6 c3B) and D6 cycle 5 sub-culture B (D6 c5B), respectively. However, W2 cycle 3 culture A at 30 µg/ml artemisinin (W2 c3A) was not cloned. The samples of these cultures were collected and stored in extraction buffer for further analysis. Artemisinin together with other conventional anti-malarials [dihydroartemisinin (DHA), artemisinin (QHS), artesunate (AS), artemether (AM), chloroquine diphosphate (CQ), lumefantrine (LUM) and piperaquine phosphate (PIP)], susceptibilities were assayed by measuring the inhibition of [3H] hypoxanthine uptake in a sterile technique [31], as described herein.

### [3H] hypoxanthine incorporation assays

*Plasmodium falciparum* growth was determined by measuring incorporation of the nucleic acid precursor [3H] hypoxanthine in the parasites genomic DNA. Test compounds were diluted in DMSO (except CQ; diluted in water) and diluted to >0.2 % DMSO. 25 µl of screening medium was added to each well (except row B) in a 96-well flat-bottom micro-culture plate (Costar^®^, Corning Incorported, NY, USA). Fifty microlitre of dissolved compounds, containing four times the highest test concentration, were added to wells of row B in duplicates. Two times serial drug dilutions were prepared using a multichannel pipette. Twenty five microlitre of drug solution were taken from wells of row B and transferred, after mixing, to wells of row C and so forth down to row H (a 64-fold range dilution). The 25 µl of diluent removed from wells of row H were discarded. Two hundred microlitre of infected erythrocytes (1.5 % haematocrit and 0.5 % parasitaemia) were then added to each well except for wells A9–A12, to which, 200 µl of uninfected RBCs (diluted in screening medium to 1.5 % haematocrit) were added as a negative control.

The plates were incubated at 37 °C in a chamber gassed by a medical grade gas mixture of 92 % N_2_, 3 % O_2_, and 5 % CO_2_ at 95 % humidity. After a 48-h incubation period, 25 µl of [3H] hypoxanthine working solution was added by pulsing to each well (0.5 µCi per well).

Plates were incubated for an additional 18 h period then frozen at negative 20 °C. After thawing, the content of the plates was harvested onto glass-fiber filter mats (A 1450-421; Perkin Elmer) using a Betaplate cell harvester (1295-004 Betaplate; Wallac Perkin Elmer) and dried in an oven.

The oven-dried filter mats were drenched in 10 ml of scintillation fluid (Beta Scintilla^®^, Perkin Elmer, Schwerzenbach, CH) in a plastic foil (1450-432; Perkin Elmer) and the [3H] hypoxanthine incorporation was measured in terms of counts per minute (cpm) using a Betaplate liquid scintillation counter (1205 Betaplate; Wallac Perkin Elmer).

The result of each well was recorded as counts per minute and expressed as percentage of the untreated (positive) control (wells A1–A8; cultures without test compound). The negative control was used for background subtraction. IC50s (drug concentration at which 50 % of [3H] hypoxanthine incorporation was inhibited compared to drug-free controls) were estimated by linear interpolation [32].

Data analysis was done using Graph Pad Prism software, version 5 (Graph Pad Software, Inc.), and the concentrations of the drugs that inhibited parasite growth by 50 % (IC50) were obtained by nonlinear regression analysis of the inhibition of uptake values and log-transformed concentration values.

### Genomic DNA isolation

The genomic DNA was extracted from 1 to 2 ml aliquots of parasite cultures by using the QIAamp mini DNA kit (Qiagen, Germany) as per the manufacturer’s specifications and eluted in 50 µl of AE elution buffer. Non-template control samples were obtained by using un-parasitized red blood cells in each extraction. The untreated parasites (i.e., no drug pressure, W2 and D6) samples, cultured for the same period of 450 days, were used as the positive controls.

### Sequencing of the *Pfmdr1*, *Pfcrt* and *Pfk13* genes

The *Pfmdr1* (PFE1150w), *Pfcrt* (MAL7P1.27), and *Pfk13* (PlasmoDB identifier PF3D7_1218300) genes were amplified from genomic DNA using polymerase chain reaction (PCR). The primers used for PCR and sequencing of the genes are shown in Table 1. The double-strand sequencing of PCR products was performed by Macrogen laboratory (Netherlands). Sequences were analysed by the BLAST programme in ncbi. Multiple nucleotide sequence alignment and analysis of the reported gene 3D7 in PlasmoDB DB and *Pfmdr1*, *Pfcrt*, and *Pfk13* sequenced *g*enes was done using the Codon code Aligner version 5.1 and MEGA 6 to identify specific single nucleotide polymorphism (SNP) combinations (standard bidirectional sequencing and reading).

**Table 1:** Primers used in PCR

Gene ID	Chromosome	Primers	Direction	PCR	size
*Pfcrt* MAL7P1.27	5	P1 (5′-gcgcgcgcatggctcacgtttaggtggag-3′)	Forward	Primary PCR	_146 bp
		P2 (5′-gggcccggcggatgttacaaaactatagttacc-3′)	Reverse		
		D1 (5′-tgtgctcatgtgtttaaactt-3′)	Forward	Nested PCR	
		D2 (5′-caaaactatagttaccaattttg-3′)	Reverse		
*Pfmdr1* PFE1150w	7	Af (5′-tgtatgtgctgtattatcaggaggaac-3′)	Forward	Primary PCR	_560 bp
		Ar (5′-aattgtactaaacctatagatactaatgataatattatagg-3′)	Reverse		
		Bf (5′-gatggtaacctcagtatc-3′)	Forward	Nested PCR	
		Br (5′-ctcctgataatacagcac-3′)	Reverse		
*Pfk13* PlasmoDB PF3D7_1218300	13	K13-1 (5′-cggagtgaccaaatctggga-3′)	Forward	Primary PCR	845 bp
		K13-4 (5′-gggaatctggtggtaacagc-3′)	Reverse		
		K13-2 (5′-gccaagctgccattcatttg -3′)	Forward	Nested PCR	
		K13-3 (5′-gccttgttgaaagaagcaga -3′)	Reverse		

### Nucleotide sequence accession numbers

The DNA sequences of representative samples showing mutant types were submitted to Gen-Bank and assigned accession numbers KU236713 and KU236714.

## Results

### Discontinuous drug pressure generates *P. falciparum* lines tolerant to increased levels artemisinin in vitro

The IC50 values of artemisinin for the parental parasite lines W2 and D6 were determined by [3H] hypoxanthine incorporation susceptibility assays [31], which were 2.85 ng/ml for W2 and 3.06 ng/ml for D6 (Table 2). As the parasites became adapted to gradually increased concentrations of artemisinin, the two lines became increasingly less susceptible to the drugs, artemisinin (QHS), dihydroartemisinin (DHA), artesunate (AS) and lumefantrine (LMF) (for D6 only), but more susceptible to CQ (Table 2).

**Table 2:** In vitro susceptibility testing of parental and resistant lines to anti-malarial drugs

Parasite line	QHS IC50	CQ IC50	PIP IC50	LMF IC50	AM IC50	DHA IC50	AS IC50
W2	2.85 ± 0.55	127.66 ± 1.96	64.18 ± 10.18	10.49 ± 0.06	3.92 ± 0.66	0.45 ± 0.16	0.60 ± 0.15
W2 c5B	9.13 ± 0.127	60.63 ± 8.62	51 ± 4.23	10.5 ± 0.27	5.44 ± 0.20	0.94 ± 0.26	2.35 ± 0.65
D6	3.06 ± 0.19	12.67 ± 0.03	44.37 ± 11.69	54.9 ± 3.99	8.74 ± 0.35	1.64 ± 0.45	1.454 ± 0.35
D6 c5B	8.26 ± 0.55	11.67 ± 2.05	55.46 ± 3.4	84.58 ± 1.64	12.26 ± 0.66	4.19 ± 1.03	7.66 ± 0.36
3D7	15.5 ± 0.95	13.36 ± 1.09	55.04 ± 0.81	54.52 ± 5.88	9.68 ± 2.17	2.78 ± 0.14	4.66 ± 0.93

W2 c5B = W2 *P. falciparum* line exposed to artemisinin, D6 c5B = D6 *P. falciparum* line exposed to artemisinin

*QHS* artemisinin, *DHA* dihydroartemisinin, *CQ* chloroquine diphosphate, *PIP* piperaquine phosphate, *AM* artemether, *AS* artesunate, *LMF* lumefantrine, *ED50* 50 % inhibitory concentration (in ng/ml; mean ± SD)

**Fig. 1:**
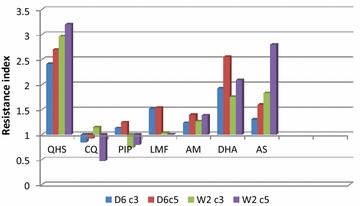
Changes in the index of resistance. Changes in the index of resistance (ratio of the IC50 for the resistant progeny to the IC50 for the parental line) to various drugs during the adaptation of the *P. falciparum* lines W2 and D6 to artemisinin. *QHS* artemisinin, *DHA* dihydroartemisinin, *CQ* chloroquine diphosphate, *PIP* piperaquine phosphate, *AM* artemether, *AS* artesunate, *LMF* lumefantrine. There was >twofold resistance increase to artemisinin and its derivatives

W2 c5B after W2 was the most tolerant strain produced from the assay described above, which was treated with artemisinin at a level that surpassed what would typically be found in plasma of malaria patients treated with artemisinin (Fig. 1). This produced W2 parasites that tolerated 50 µg/ml artemisinin for 48 h.

**Fig. 2:**
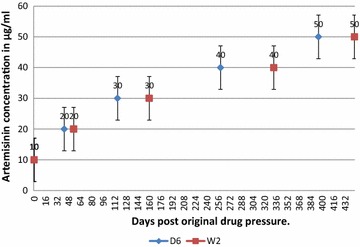
*Plasmodium falciparum* lines discontinuous artemisinin pressure. W2 and D6 *P. falciparum* lines were exposed to a discontinuous artemisinin pressure up to 50 µg/ml within a period of 450 days

The adaptation pattern observed was similar, a marked reduction in artemisinin susceptibility when the parasites became adapted to 50 µg/ml (Fig. 2), a phenotype manifested the selected progenies progressing normally through the erythrocytic stages of the lifecycle, with no substantial differences in growth from the parental lines in the absence of artemisinin pressure.

### Resistant phenotype was due to polymorphism in *Pfmdr1* gene

Key polymorphic regions of the *Pfmdr1* gene that covered codons 86 and 184 were sequenced successfully in the parental and resistant progenies. Mutations N86Y was observed in the parental W2 parasite lines, while D6 parasites had “wild-type” *Pfmdr1*. Interestingly the parental parasites and their respective progenies had Y184 codon but I185K mutation was reported as a novel polymorphism. The amino acid codons at these positions are shown in Table 3.

**Table 3:** *Pfmdr1* and *Pfcrt* allelic types in parental and resistant *P. falciparum* lines

Parasite line	*Pfmdr1* Amino acid position			*Pfcrt* Amino acid positions				
	86	184	185	72	73	74	75	76
W2	Y	Y	I	C	V	I	E	T
W2 c3B	Y	Y	K	C	V	I	E	T
W2 c5A	Y	Y	K	C	V	I	E	T
W2 c5B	Y	Y	K	C	V	I	E	T
D6	N	Y	I	C	V	M	N	K
D6 c3A	N	Y	K	C	V	M	N	K
D6 c3B	N	Y	K	C	V	M	N	K
D6 c5A	N	Y	K	C	V	M	N	K
D6 c5B	N	Y	K	C	V	M	N	K

D6 c3A, D6 c3B, D6 c5A, D6 c5B = D6 *P. falciparum* line exposed to artemisinin. W2 c3B, W2 c5A, W2 c5B = W2 *P. falciparum* line exposed to artemisinin

### Codons 72–76 of *Pfcrt*

The wild-type *Pfcrt* allele, encoding CVMNK at codons 72–76 of the *crt* protein was confirmed as present in both D6 (one sample of the one sequenced, D6 isolate) and its tolerant progenies (four samples of the four sequenced, D6 c3A, D6 c3B, D6 c5A and D6 c5B), but W2 (one sample of the one sequenced, W2 isolate) and its subsequent progenies (three samples of the three sequenced, W2 c3A, W2 c5A and W2 c5B) harboured the CVIET allele.

### *Pfk13* propeller

The changes in amino acid sequences at codon C580Y, R539T, I543T, and Y493H as has been reported by Ariey et al. [5], were not detected in either parent lines W2 and D6 or their artemisinin tolerant progenies and all the coding sequences were “wild type”.

## Discussion

There are several drawbacks to ACT as the current strategy, despite its success over the last decade. The WHO [33] currently endorses five combinations artemether-lumefantrine, dihydroartemisinin–piperaquine phosphate, artesunate-amodiaquine, artesunate-mefloquine, and artesunate–pyronaridine. ACT is a highly used anti-malarial, creating a substantial selection advantage for parasites to evolve resistance independently with a precipitous decline in the artemisinin susceptibility.

In 2014, the number of courses treatment using various artemisinin-based combinations increased from 11 million in 2005 to 337 million [34], but the mechanisms by which artemisinin act is still under investigation. Deducing molecular genetics on its resistance is paramount for determining treatment strategies, mapping the spread of resistance and guiding elimination [27, 35, 36]. For the new tools for resistance surveillance to be validated a better understanding of the mechanisms of artemisinin resistance is needed. These tools are essential in guiding national treatment policies and helping in designing and deploying new drug combinations that may counter the emergence and spread of resistance.

In the present study, the discontinuous method of inducing resistance/tolerance to artemisinin in W2 and D6 lines of *P. falciparum* was used [30]. In agreement with other study [36], the in vitro developed artemisinin-tolerant parasites could not grow permanently under artemisinin pressure. Hence resistance to artemisinin is different from resistance to other conventional anti-malarial drugs, such as amodiaquine. The culmination of in vitro resistance as used here in, is the ability for parasites to survive pharmacologically relevant exposure of artemisinin (levels considered clinically relevant in vivo) over many generations and by major increases in the IC50. After conducting the [3H] hypoxanthine incorporation susceptibility assays, it was found that resistant parasites showed the greatest tolerance for artemisinin (W2 c5B and D6 c5B). The study hypothesized a reduced susceptibility to this drug, because it was used to induce resistance in these strains. The discontinuous drug pressure selection scheme is supposedly to have been the key to success in selecting the tolerant lines [30]. After adapting, the parasites were able to tolerate as much as 50 µg/ml of artemisinin for 48 h without noticeable changes in growth rate or morphology. The results also showed resistant parasites generally had decreased susceptibility to artemisinin, but were more susceptible to CQ, which is in agreement with other studies [7, 37, 38]. There was a significant degree of cross-resistance to various other artemisinin derivatives, including artemether, artesunate, and dihydroartemisinin which were not utilized in the generation of resistance in those particular lines. This highlights that, it is possible to select resistance in a drug-sensitive background (D6) African isolate, in antagonism to conclusion from other studies in which it was stated that multiple resistance phenotypes must pre-exist [39, 40].

These artemisinin-tolerant parasites provided a unique resource for the study of the artemisinin resistance mechanism(s) and modes of action based on analysis of SNPs in *Pfmdr1*, *Pfk13* and haplotypes in *Pfcrt*. The rationale for considering using these three genes were (i) that a relationship between SNPs in *Pfmdr1* and ACT has been reported, suggesting that *Pfmdr1*-N86 or -184F was selected in reinfections after artemether-lumefantrine treatment; and moreover, *Pfcrt*-76K and *Pfmdr1*-N86 were selected by artemether-lumefantrine in recurrent infections [20]. *Pfmdr1* and *Pfcrt* were selected for chloroquine resistance and they also both have been reported to be associated with in vitro reduced susceptibility to artesunate and amodiaquine [41]. (ii) Genome association studies strongly suggested a locus on *P. falciparum* chromosome 13 gene PF3D7_1343700 (bases 1,724,817–1,726,997, PlasmoDB 11.1) to artemisinin resistance and mutations discovered in the *Pfk13* were strongly linked to artemisinin resistance [5].

It is well known that *Pfmdr1* is associated with drug resistance in Asia [7, 38]. *Pfmdr1* encode protein of the ATP binding cassette (ABC) transporter superfamily encoding P-glycoprotein homologue-1 [42], which are localized to the parasite food vacuole membrane or plasma membrane and can decrease intracellular drug accumulation by pumping out drugs.

SNPs at codons 86, 184, and 1246 of the *Pfmdr1* gene has been suspected to be the markers of changes in parasite susceptibility to various drugs, including ACT [43]. This shows that monitoring of changes in prevalence of SNPs in the *Pfmdr1* gene may serve as an early warning tool to selection of *P. falciparum* tolerant/resistant to ACT [44].

In the two artemisinin-tolerant parasite lines used in the present study, the decrease in parasite susceptibility to the drug was associated with substitution of mutation at codon Y184F for I185K. This conforms with other studies where sequence variation and increase in copy number in *Pfmdr1* were reported to interfere with the parasite susceptibility to dihydroartemisinin [7] and artemisinin [9]. This is because; the mutation at codon Y184F was not detected in the two studies. However, other studies have shown a relationship between SNPs in *Pfmdr1* and ACT drugs, in that *Pfmdr1*-N86 or -184F was selected in reinfections and *Pfcrt*-K76 and the *Pfmdr1*-N86 in recurrent infections after artemether-lumefantrine treatment [20].

It is established that ACT can select for particular alleles of two genes, *Pfmdr1* and *Pfcrt*, which are firmly associated with resistance to the 4-aminoquinolines chloroquine and amodiaquine. The *Pfcrt* gene that encodes a 424-amino acid integral 10 trans-membrane domains protein is a transporter present in the parasite digestive vacuole membrane.

In this work, it was observed that *Pfcrt*-CVMNK were the favoured haplotypes. This can be attributed to compensatory mutation at C350R in a different study, which was shown to explain the seemingly discordant chloroquine response in *Pfcrt*-CVIET haplotype [45]. This may suggest the benefit of the two wild-type genes, *Pfmdr1*-N_86_ Y_184_ and *Pfcrt*-CVMNK, was enhanced by the presence of artemisinin.

Ariey et al. [5] linked artemisinin resistance in *P. falciparum* to slow clearance rates in patients which he strongly associated with single point mutations in the “propeller” domain of the *P. falciparum kelch* protein gene on chromosome 13 (*kelch13*) (PlasmoDB identifier PF3D7_1218300). 17 mutations in the propeller region are linked to artemisinin drug resistance. The *Pfk13* gene is well conserved across *Plasmodium* species and is thought to mediate protein–protein interactions [5].WHO included *Pfk13* mutations (Y493H, I543T, R539T, C580Y and M476I) in the new working definition for suspected artemisinin resistance in August 2014 [46]. The present study did not detect any polymorphisms reported elsewhere to reduce *P. falciparum* susceptibility in vivo in the coding sequences of the *Pfk13* gene.

Although it is clear that *Pfmdr1* may play a significant role in artemisinin resistance, some studies also demonstrated that changes in susceptibility to mefloquine or halofantrine could be achieved without changes in the expression level or allelic type of *Pfmdr1* [47, 48], suggesting a possible involvement of other genes and/or mechanisms of sustaining resistance in these parasites, including transient changes in gene expression.

Other work has reported lack of mutations in *Pfmdr1* and *Pfcrt* in parasites cultured for 5 years under artemisinin derivatives in vitro, and *Pfmdr1* amplification was not observed. Further, four mutations were discovered in *Pfk13* (Y493H, I543T, R539T and C580Y). In the same study, 17 single K 13-propeller mutations were detected in naturally circulating parasites in Cambodia [5]. However in the present study, there was *Pfmdr1* novel mutation at codon I185K and no non-synonymous mutations in *Pfk13* gene. This difference can be attributed to the shorter period (450 days) of artemisinin exposure.

## Conclusion

ACT is the critical tool for controlling the world’s most important parasitic disease; malaria. This necessitates research on artemisinin resistance markers for epidemiological surveys in malaria endemic regions. This work has demonstrated that resistance to artemisinin can be induced in *P. falciparum* in vitro. Although long-term stability studies at high levels of drug exposure have not been completed; this data suggest that stable artemisinin tolerance was selected for by multiple pulse exposures to drug over time. This is reflected in the substitution of *Pfmdr1* Y184F mutation for I185K. Clearly, more studies are required to identify the mechanism(s) by which resistance to artemisinin emerges and to find molecular genetic markers that can be used for epidemiological studies to track the possible emergence of artemisinin resistance.

## Recommendations

High artemisinin resistant lines should be selected and probably to a line which can grow continuously under artemisinin pressure. Thereafter, the investigations into artemisinin resistance markers mainly targeting the implicated genes *Pfk13*, *Pfmdr1* and *Pfubcth* associated with reduction in drug efficacy should be performed. Structured studies that mitigate confounding factors such as multiple clonal infections in a variety of crossing experiments should be investigated in vivo. This is because in the laboratory, the effect of the mutation is determined in only one genetic background but a wide variety of backgrounds are found in the field due to recombination.
